# Comparative Perceptions of Fluoride Toxicity in Oral Hygiene Products: Insights from the General Population and Healthcare Professionals

**DOI:** 10.3390/clinpract14050146

**Published:** 2024-09-05

**Authors:** Marija Badrov, Lidia Gavic, Ana Seselja Perisin, Davor Zeljezic, Jasen Vladislavic, Ema Puizina Mladinic, Antonija Tadin

**Affiliations:** 1 Department of Restorative Dental Medicine and Endodontics, University of Split School of Medicine, 21000 Split, Croatia; mb91802@mefst.hr (M.B.); atadin@mefst.hr (A.T.); 2 Department of Pharmacy, University of Split School of Medicine, 21000 Split, Croatia; ana.seselja.perisin@mefst.hr; 3 Division of Toxicology, Institute for Medical Research and Occupational Health, 10000 Zagreb, Croatia; dzeljezi@imi.hr; 4 Department of Pulmonology, Clinical Hospital Centre Split, 21000 Split, Croatia; jvladislavic4@gmail.com; 5 Department of Maxillofacial Surgery, Clinical Hospital Centre Split, 21000 Split, Croatia; e.puizina@gmail.com

**Keywords:** fluoride, knowledge, healthcare, oral hygiene, toxicity

## Abstract

Background: The safety of oral hygiene products is a growing concern, particularly regarding the toxicity of specific ingredients used in their formulations. The purpose of this study was to evaluate the knowledge and attitudes of dentists, physicians, pharmacists, and the general public regarding ingredients in oral hygiene products, especially fluoride. Additionally, this study aimed to identify which ingredients may exhibit potential toxicity based on historical records of any adverse effects being induced by a material/component. Methods: A self-administered questionnaire was used in an online cross-sectional observational study to collect data on sociodemographic characteristics, knowledge of fluoride in dental medicine, fluoride usage practices in oral hygiene products, opinions on ingredient toxicity in oral hygiene products, and personal experiences of adverse reactions to products and their components. The collected data underwent descriptive and regression analyses to reveal patterns and relationships within the dataset. Results: The study found a moderate overall knowledge level regarding fluoride usage in dentistry among participants (Md = 5.00, IQR 2.50–7.00). Healthcare professionals exhibited significantly higher knowledge scores compared to the general population (*p* ≤ 0.001), with dental professionals displaying the highest scores. Regarding concerns about the usage of fluoride, the majority of respondents (77.0%) did not express any concerns. Minor concerns included the risk of ingestion (6.0%) and dental fluorosis (4.6%). Among the other ingredients in oral hygiene products, respondents named alcohol as the most toxic ingredient (70.3%), followed by artificial colors (53.1%), artificial sweeteners (50.4%), and parabens (50.1%). It is noteworthy that the majority of participants (61.6%) stated that they had never experienced any side effects associated with the use of oral hygiene products. Conclusion: This study underscores disparities in fluoride knowledge between healthcare professionals and the general population in Croatia, with dental experts exhibiting a superior understanding. Despite lingering misconceptions about fluoride content and potential toxicity, the majority of participants acknowledge its oral health benefits and use fluoride products regularly.

## 1. Introduction

Fluorides are extensively utilized in oral hygiene products due to their well-documented efficacy in preventing dental caries. Their preventive action is mediated through multiple mechanisms: reducing bacterial metabolism, which decreases acid production and subsequent demineralization; promoting remineralization; and altering tooth structure to reduce solubility [1,2]. These compounds are incorporated into various oral care products, including toothpaste, mouthwash, and dental materials [3]. Effective caries prevention necessitates diligent oral hygiene practices, including bi-daily brushing with fluoride toothpaste for at least two minutes [4]. Additionally, water fluoridation, which introduces controlled fluoride levels into public water supplies, contributes to community-wide dental health [5]. However, the efficacy of this method can be inconsistent due to varying water consumption and the use of water filters. Fluoride in oral products is available in forms such as sodium fluoride, sodium monofluorophosphate, tin fluoride, and amine compounds [6]. Toothpaste typically contains 1000 to 1500 ppm of fluoride, with higher concentrations often requiring a prescription due to the potential risks of dental fluorosis [4].

However, it is important to emphasize that along with all the benefits of fluorides, their use also has associated risks. One of the primary concerns related to the use of fluoride is the occurrence of dental fluorosis, which usually occurs when excessive fluoride is ingested during enamel formation in developing teeth. In some severe cases, this develops into skeletal fluorosis, characterized by increased bone density visible on X-rays but associated with fragility [3,7]. Therefore, young children should be supervised when using topical products to prevent possible fluorosis, as swallowing even small amounts during tooth development can contribute to its occurrence [7]. Highlighting the significance of healthcare providers and dental professionals is crucial in advocating for safe toothpaste usage, especially among vulnerable groups such as young children, who are at a higher risk of accidental ingestion [8]. In Europe, although there are clear guidelines for fluoride application in children, there is a lack of standardized recommendations for adults, suggesting that there is still a need for development in this area to improve preventive dental care in all age groups [9,10].

Other oral hygiene ingredients may likewise carry potential toxicity risks. Alcohol in mouthwashes may lead to burning sensations and is not recommended for certain groups such as infants and individuals with an alcohol addiction. While it has been associated with adverse effects like xerostomia, evidence linking it to oral cancer is inconsistent [11]. Sodium lauryl sulfate (SLS), a common toothpaste detergent, can damage oral tissues and increase mucosal desquamation [12,13]. Ingredients like parabens and sodium benzoate have potential adverse effects on oral mucosa, including carcinogenicity and allergic reactions [14]. Consumers and oral health professionals must be aware of these risks to ensure safe product usage.

Several studies worldwide have investigated the knowledge and perceptions of fluoride among both healthcare professionals and the general population [15,16,17,18,19,20,21,22,23,24,25,26,27,28,29,30,31,32,33]. It was noted that dental professionals consistently demonstrated a deeper understanding of this topic compared to other groups. This observation finds support in research such as the population-based study on awareness of fluoride benefits and risks among doctors in China [15], as well as comparisons of oral health knowledge between dental healthcare professionals and the general public in the UK [16]. Similarly, a study from Saudi Arabia showed that dental health workers exhibit positive attitudes and knowledge regarding fluoride as a preventive measure [17]. On the other hand, studies have also pointed out inadequate awareness regarding fluoride among other health professionals, such as medical and paramedical students in a study performed in Mangalore [18] and health professionals in Saudi Arabia [19]. Additionally, poor awareness regarding the benefits of fluoride was noted among half of the pharmacists in a study conducted in India [20].

Understanding the attitudes and knowledge regarding fluoride among healthcare professionals and patients is crucial, as these factors directly influence the adoption of evidence-based oral health practices [15,29,34]. Healthcare professionals, including pharmacists and physicians, play a vital role in educating patients and recommending preventive measures, such as fluoride use. Although dentists are primarily responsible for oral health, pharmacists and physicians often serve as the first point of contact for patients with health concerns and questions about over-the-counter dental products. Therefore, they must be knowledgeable about the benefits and potential risks of fluoride, which is widely used and carries significant health implications. If healthcare professionals’ knowledge is incomplete or their attitudes are negative, it could hinder the promotion of fluoride-based interventions. Conversely, well-informed professionals can offer accurate advice on the safe use of fluoride products, recognize symptoms of fluoride overexposure, and understand the toxicology of other common ingredients in oral hygiene products [15,35,36,37]. Similarly, patient attitudes and knowledge are critical in determining their willingness to follow these recommendations. Evidence shows that individuals who are well-informed about fluoride’s preventive benefits are more likely to engage in recommended oral hygiene behaviors, while negative perceptions or misinformation may lead to the avoidance of fluoride-based products, potentially compromising oral health [21,38,39,40,41,42]. This comprehensive approach to healthcare not only supports better oral health outcomes but also enhances overall patient care, benefiting both individual patients and the wider community [35,36,37].

This study investigates the perception of fluoride toxicity and the toxicity of oral hygiene products among health professionals and the general population in Croatia, filling a significant research gap by investigating a topic that has not been studied in this country before. Our research aims to assess knowledge and attitudes towards fluoride among dental, medical, and pharmaceutical professionals and the general public and to investigate their views on fluoride and other ingredients in oral hygiene products, the perceived toxicity of these ingredients, and their experiences of adverse effects. The research question aims to identify differences in the knowledge and attitudes of these groups and to understand how these perceptions influence their views on the risks and benefits of fluoride. The null hypothesis is that there is no significant difference in knowledge and attitudes regarding the toxicity of fluoride and oral hygiene products between these occupational groups and the general population. By bridging the gap between scientific evidence and public understanding, this study aims to improve health outcomes and promote evidence-based oral care practices.

## 2. Materials and Methods

### 2.1. Study Design and Population

This cross-sectional study was based on a convenience sample and was c1onducted between February and April 2024 in the Department of Restorative Dentistry and Endodontics at the School of Medicine, University of Split, Croatia. Data collection was conducted by means of an online survey using a Google Form (Google Forms, Google, Mountain View, CA, USA). The study was approved by the Ethics Committee of the University of Split, ensuring compliance with established guidelines and regulations, including the World Medical Association’s Declaration of Helsinki. Participation was entirely voluntary, and respondent anonymity was maintained throughout the survey.

This study included the adult population of Croatia. Respondents were divided into four categories, including dental, medical, and pharmaceutical professionals and patients from the departments involved in this study. Respondents were recruited by distributing an online survey link through available email addresses. In the introductory part of the survey, the respondents were asked to forward the questionnaire to all interested colleagues using the snowball method. Participants received two reminders, with a two-week interval between each reminder. The minimum sample size required for successful completion of the study (N = 385) was calculated using the Sample Size Calculator software (Inc.RaoSoft^®^, Seattle, WA, USA). The calculation was based on the number of adult populations in the Republic of Croatia according to the latest census data (N = 3,223,679) from 2022, with a confidence interval of 95 and a margin of error of 5 [43]. 

The inclusion criteria for the study required respondents to be residents of Croatia, be at least 18 years old, and have access to the Internet and an email address. Specifically, adult patients of the Department of Restorative Dentistry and Endodontics, as well as dentists, physicians, and pharmacists practicing in Croatia, were included in this study, provided they were willing to participate in an online survey. Participants also had to be actively practicing in their respective professions. Exclusion criteria included minors, people residing outside of Croatia, individuals with incomplete data, and participants who did not have access to the Internet or an email address. Additionally, people who were not actively working in their profession were excluded.

### 2.2. Questionnaire

The data used in this study were collected through a questionnaire that was developed based on several surveys with similar topics [15,16,17,18,19,20,21,22,23,24,25,26,27,28,29,30,31,32,33]. This survey was developed in the Croatian language by a professor of endodontics and restorative dentistry (A.T.), a professor of pediatric dentistry (L.G.), and a dental student (M.B.). It was then pilot tested with 25 individuals who met the inclusion criteria but were not part of the final study sample. These participants assessed the survey’s usability and technical performance. Feedback from this pilot test led to revisions aimed at improving the clarity and comprehensibility of the survey questions. The final version of the survey was subsequently approved by two medical professionals (E.P.M. and J.V.) and a professor of pharmacy (A.S.P.). The fluoride knowledge questionnaire demonstrated acceptable reliability, with a Cronbach’s alpha coefficient of 0.806 based on pilot data. The survey required approximately 10 min to complete.

The questionnaire comprised 63 questions organized into seven sections, starting with the first one (Q1–Q6), which aimed to collect socio-demographic and occupational data from the respondents. This included information on gender, age, education level, employment status, field of education/occupation, and socio-economic status. The second section (Q7–Q13) included questions on respondents’ attitudes towards fluoride use and application. This included specific questions aimed at ascertaining respondents’ awareness of the purpose of fluoride and the types of fluoride they use. The third part of the questionnaire focused on the assessment of knowledge about the effects of fluoride (8 questions, Q14–Q21), where respondents chose between three options: “Yes”, “No” or “I do not know”. A scoring system was used where correct answers (“Yes”) earned one point and incorrect answers received zero points. Each respondent’s total score, based on the number of correct answers, provided a quantitative measure of their knowledge. According to Bloom’s taxonomy cut-off points, scores were categorized as follows: 6.4 to 8 points (80–100%) indicated good knowledge, 4.8 to 6.3 points (60–79%) indicated moderate knowledge, and below 4.9 points (<60%) indicated poor knowledge [44]. Respondents could score a maximum of 8 points in this section of the questionnaire assessing their level of knowledge. In the fourth section (Q22–Q36), participants were presented with a summary of certain ingredients in oral hygiene products, including fluorides, alcohol, parabens, and others, and were asked to assess their toxicity. The fifth section (Q37–Q46) collected information on any side effects that participants had experienced when using oral hygiene products, while the sixth section (Q47–Q54) asked participants to name the oral hygiene products that they associated with potential damage to the oral mucosa. The seventh and final section of the survey consisted of nine questions (Q55–Q63) focusing on participants’ primary concerns regarding the impact of fluoride on human health.

### 2.3. Data Analysis

The Kolmogorov–Smirnov test was used to assess the normality of the data distribution. Descriptive statistics provided summaries of the data, including frequencies and percentages for categorical variables and means or medians with standard deviations or interquartile ranges for continuous variables. To analyze the relationships between fluoride knowledge and sociodemographic and occupational factors, Mann–Whitney or Kruskal–Wallis tests were employed. Chi-square tests were used to compare categorical variables across different groups (the general population, dentists, physicians, and pharmacists). Statistical significance was determined at *p* < 0.05. Data analysis was performed using SPSS Version 26 (SPSS, IBM Corp, Armonk, NY, USA).

## 3. Results

Table 1 summarizes the socioeconomic and occupational characteristics of the study participants and their correlation with fluoride knowledge. A total of 1129 adult respondents participated in this study. The majority (76.9%) of participants were female, with just over half (53.0%) in the under-30 age group. Factors such as sex, age, education level, employment, and socioeconomic status showed significant influence on the level of knowledge about fluorides (*p* < 0.05). Women demonstrated significantly higher knowledge than men (*p* = 0.002). The highest fluoride knowledge was observed among dental professionals compared to other healthcare professionals and the general population (*p* ≤ 0.001), while pharmacists showed better fluoride knowledge in comparison to medical professionals and the general population.

Table 2 illustrates participants’ attitudes and practices regarding fluoride use and application. More than three-quarters of respondents were aware of the anti-cariogenic effects of fluoride (N = 887, 78.6%). Those who supported these notions also had statistically significantly higher fluoride knowledge scores (*p* ≤ 0.001). The majority of these respondents used fluoridated toothpaste (68.2%) in their daily toothbrush routine. The majority of respondents from this study, however, did not use fluoridated mouthwash (68.7%) or any other source of fluoride (60.0%).Participants were unaware of the fluoride concentration in toothpaste (64.0%) and the most commonly used chemical compound of fluoride (59.7%). The chi-square test was used to examine the differences between the study groups, confirming statistically significant differences in awareness of the effect of fluoride on reducing the incidence of caries and the use of fluoridated toothpaste in daily routines (*p* ≤ 0.001). However, there were no significant differences in the most commonly used forms of fluoride in toothpaste, fluoride content in ppm, the use of fluoridated mouthwash, and other fluoride-containing products (*p* < 0.05).

Table 3 illustrates the distribution of correct responses to questions regarding fluoride knowledge. Respondents demonstrated a moderate level of knowledge, with the median knowledge score concerning fluoride at 5.00 (IQR 2.50–7.00, min 0, max). Just over half of the participants (57.0%) possessed a level of knowledge at or above the median. Notably, those who were either educated or employed in the dental field exhibited higher knowledge level scores (Md 7.00, IQR 6.26–6.65, min 0, max 8). The most frequently correctly answered question (N = 826, 73.2%) pertained to the statement “Brushing twice a day with fluoride toothpaste lowers the risk of dental caries”. On the other hand, only 43.7% of the participants demonstrated an understanding of fluoride’s role in preventing early dental caries by reducing bacterial metabolism. For all the questions assessing fluoride knowledge, the chi-square test revealed a statistically significant difference between the groups (*p* ≤ 0.001).

Table 4 provides a summary of specific ingredients of oral hygiene products and assesses their toxicity as evaluated by the participants. The participants regarded alcohol (70.3%) as the most toxic ingredient, followed by artificial dyes (53.1%), artificial sweeteners (50.4%), and parabens (50.1%). Fluorides were labeled as toxic by 30.9% of the participants. The chi-square test confirmed a statistically significant difference (*p* ≤ 0.001) between the four study groups in the assessment of all oral hygiene product ingredients among the participants.

**Figure 1:**
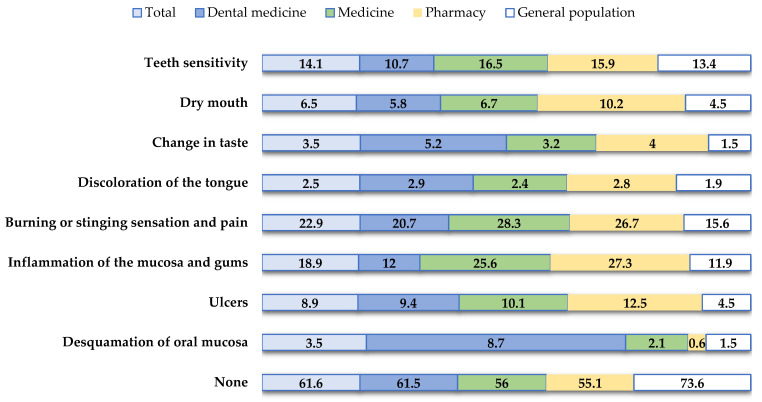
Participant experiences of side effects associated with oral hygiene product usage.

Figure 1 provides information regarding the side effects experienced by participants while using oral hygiene products. The majority of them (N = 695, 61.6%) have never experienced any adverse reactions. Conversely, 22.9% of them reported experiencing burning or stinging sensations, and 18.9% experienced inflammation of the oral mucosa.

**Figure 2:**
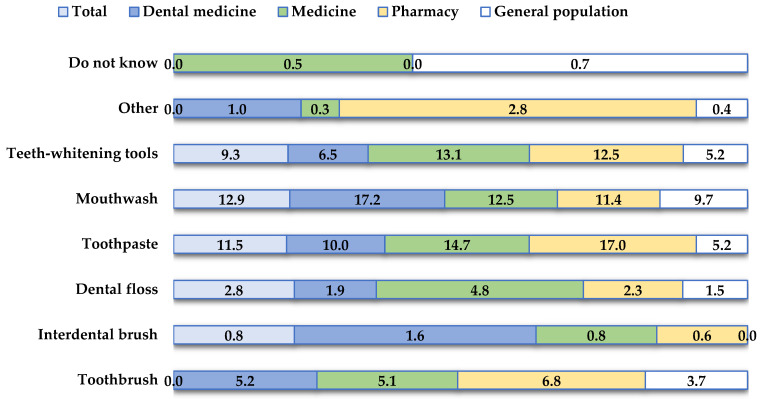
Oral hygiene products associated with potential oral mucosal damage as reported by participants.

Figure 2 shows the oral hygiene products that participants reported to be associated with possible damage to the oral mucosa. Respondents indicated that they had experienced damage to the oral mucosa when using mouthwash (N = 146, 12.9%) and toothpaste (N = 130, 11.5%).

**Figure 3:**
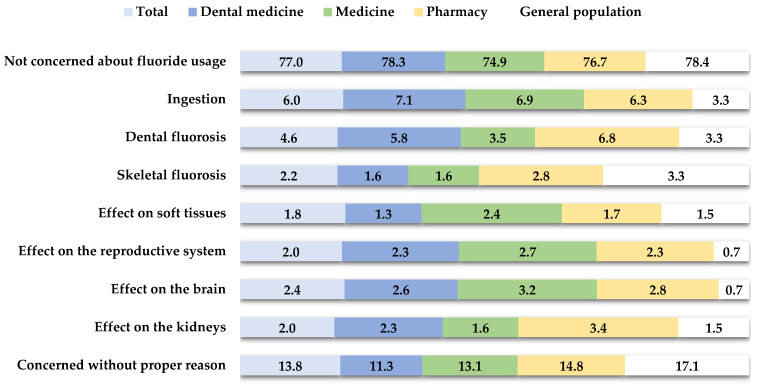
The distribution of primary concerns regarding fluoride usage among participants.

Figure 3 summarizes the participants’ main concerns regarding the use of fluoride. The majority of them (77.0%) did not express concern about the use of fluorides. In addition, 13.8% of respondents indicated that they were concerned about the use of fluoride but could not specify the exact reason. Lesser concerns included the risk of ingestion (N = 67, 6%) and dental fluorosis (N = 52, 4.6%).

## 4. Discussion

Fluoride strengthens tooth enamel, reduces dental caries, and supports overall oral health. It is widely utilized in water fluoridation, toothpaste, and professional treatments [3]. Despite its established benefits, misconceptions about fluoride persist [45]. This study aimed to evaluate the understanding of fluoride among healthcare professionals and patients and to investigate misconceptions related to its toxicity in oral hygiene products. The key findings of this study reveal that dental professionals possess a comprehensive understanding of fluoride, whereas other healthcare professionals and the general public exhibit only a moderate level of knowledge. Despite this moderate level of knowledge, there is either a lack of concern or minimal concern regarding fluoride use, and no significant correlation is found between the level of knowledge and the degree of concern about fluoride’s potential risks.

This study observed a moderate level of knowledge about fluoride among participants. Healthcare professionals scored notably higher than patients (*p* ≤ 0.001), with dental professionals achieving the highest scores. Factors like gender, age, and socioeconomic status did not significantly influence knowledge levels. Dental professionals consistently showed a deeper understanding of fluoride compared to other groups. This trend is reflected in studies such as a population-based study on awareness of the benefits and risks of fluoride among doctors in China [15] and comparisons of oral health knowledge between dental professionals and the general public in the UK [16]. In a study conducted in Saudi Arabia, dental professionals also showed a positive attitude and knowledge about fluoride as a preventive measure [17]. This observation can be attributed to the fact that individuals working in dentistry generally have extensive knowledge about oral health and the role of fluoride. This is largely attributed to their specialized training and professional experience in dentistry [46]. Conversely, some studies have highlighted the lack of awareness of fluoride among other health professionals, including medical and paramedical students in a study conducted in Mangalore [18] and health professionals in Saudi Arabia [19]. In a study carried out in India [20], half of the pharmacists surveyed were also insufficiently informed about the benefits of fluoride. 

The majority of participants in this study were aware of the role of fluoride in caries prevention (N = 887, 78.6%) and the importance of using fluoridated toothpaste (n = 848, 75.1%). The participants who agreed with these ideas also had statistically significant fluoride knowledge scores (*p* ≤ 0.001). This result was also observed in a study among residents of Jeddah City in Saudi Arabia, which investigated attitudes, practices, and knowledge about the use of fluoridated toothpaste [21]. In this study, 68.2% of participants used fluoridated toothpaste in their daily oral hygiene. However, about one-fifth of the participants (N = 220, 19.5%) were unsure whether their toothpaste contained fluoride, while 12.3% of them used non-fluoridated toothpaste. The participants also expressed uncertainty about both the fluoride concentration in their toothpaste (64.0%) and the most commonly used chemical compound of fluoride (59.7%). Some studies, such as those among Indian adolescents [20] and outpatients at Rama Dental College [23], reported a higher proportion of uncertainty about the fluoride content in toothpaste among participants, with rates of 57.5% and 22.5%, respectively. A study conducted in the Croatian general population also reported that 80.0% of adults were uncertain about this aspect [46]. In addition, almost half of the outpatients at Rama Dental College reported using non-fluoridated toothpaste [23]. The use of other sources of fluoride, particularly mouthwash, was very low in this study. Overall, 68.7% of the participants stated that they did not use fluoridated mouthwash as part of their oral hygiene routine. This trend has also been observed in other studies, including research among health professionals in Saudi Arabia [19] and a study among students in Malaysia [24].

The great majority of the participants in this study (N = 826, 73.2%) agreed that brushing one’s teeth twice a day with fluoride toothpaste reduces the risk of dental caries. The importance of tooth brushing and the caries-preventive action of fluoride was also recognized by healthcare workers in other studies, such as healthcare workers in a special children’s center in Riyadh City [25], dental students in Brazil [26], and physicians in Tehran [27]. However, research conducted among Indian adolescents revealed that merely 12.5% of its participants were familiar with the caries-preventive benefits of fluoride [22]. In this study, less than half (43.7%) of the participants demonstrated an understanding of fluoride’s role in preventing early dental caries by reducing bacterial metabolism. On the contrary, a study conducted among dentists in Texas revealed a significantly higher percentage (84.0%) of participants supporting this assertion [28]. Fluoride’s effects, whether beneficial or harmful, depend on factors such as concentration, frequency of use, and age [6]. In this study, 59.4% of participants acknowledged this variability. Dental professionals demonstrate a higher level of awareness, with 91.0% of oral health care providers in Kuwait [29] recognizing these factors, compared to 41.5% of outpatients at Rama Dental Clinic [23]. Children must use age-appropriate amounts of toothpaste to minimize fluoride ingestion: a smear for those under 3 years and a pea-sized amount for those 3 years and older who can reliably spit [47]. However, only 52.6% of participants in this study were aware of these guidelines. Brazilian dental students exhibited superior knowledge [26], reflecting the generally greater awareness among dental professionals. Additionally, a comparison between dental and medical professionals and the general public revealed significant differences in knowledge regarding appropriate fluoride concentrations for children [16].

Although fluoride is generally considered safe and effective when used properly in dental care products and drinking water, it is important to note that excessive intake can lead to toxicity and adverse health effects [3]. Surprisingly, a significant proportion of participants in this study (30.9%, N = 349) expressed the belief that fluoride contained in oral hygiene products was toxic, leading 12.3% of them to opt for non-fluoridated toothpaste. A study conducted in Jeddah City, Saudi Arabia, shed light on residents’ attitudes towards fluoridated toothpaste. Of those who preferred non-fluoridated toothpaste, almost half (47.6%) cited concerns about perceived toxicity as a reason for rejecting fluoridated variants [21]. According to participants’ assessments in this study, the greatest threat to health in oral hygiene products was perceived to be alcohol by 70.3% of the participants. This is particularly important considering that alcohol in mouthwashes is associated with several adverse effects, including xerostomia, a burning or sore sensation, and the potential risk of ingestion by children. However, there is weak, inconsistent, and even contradictory evidence in the literature for the purported association between alcohol-containing mouthwashes and oral cancer [11]. Half of the participants (N = 566, 50.1%) in this study associated parabens and 24.5% associated sodium benzoate with potentially toxic effects on the oral mucosa. These preservatives in oral hygiene products are known to have some adverse effects. Paraben is considered carcinogenic in high concentrations, while sodium benzoate can cause allergic reactions [14]. Despite sodium lauryl sulfate being recognized as one of the most cytotoxic ingredients in toothpaste [12,13], only 31.3% of participants in this study identified it as toxic.

Oral hygiene products play a crucial role in maintaining oral health by effectively fighting plaque and preventing oral diseases. However, it is important to note that while these products offer numerous benefits, they may also have some potential adverse effects [11,12,13,14]. In this study, the majority of participants (N = 695, 61.6%) reported that they had never experienced adverse effects from oral hygiene products. Conversely, 22.9% of respondents reported burning or stinging sensations, while 18.9% experienced inflammation of the oral mucosa. Respondents also associated this damage to the oral mucosa with the use of mouthwash (12.9%) and toothpaste (11.5%). Mouthwashes containing alcohol may cause adverse effects in certain individuals, such as a burning sensation, and may be contraindicated for certain groups such as young children, individuals with an alcohol addiction, and patients with mucosal lesions [11]. In addition, sodium lauryl sulfate (SLS), the most commonly used detergent in toothpaste, has been reported to have harmful effects on the oral soft tissue. Studies have shown that SLS in toothpaste significantly increases the incidence of oral mucosal desquamation compared to toothpastes containing other detergents [12,13]. Therefore, it is crucial for both consumers and oral health professionals to be aware of these potential risks to ensure the safe and effective use of oral hygiene products.

Fluoride helps to prevent dental caries by reducing demineralization, inhibiting microbial growth, and promoting enamel remineralization through fluorapatite formation [5]. Despite their positive effects on oral health, fluorides can also have negative effects on health. Systemic ingestion of fluorides and accidental ingestion of fluoridated toothpaste can lead to dental fluorosis [3]. Dental fluorosis is caused by excessive intake of fluoride during tooth formation and primarily results in a cosmetic disorder characterized by mottled teeth. In severe cases, it can progress to skeletal fluorosis, in which the bones become radiologically dense yet brittle [5]. Concern about fluoride and its potential harms was low in this study, with 77% of participants not worried about its use. Notably, 13.8% expressed concern without specifying reasons. Similar findings were noted among Chinese physicians [15]. Only 6% cited fluoride ingestion as a concern. This mirrors results from studies in the USA [30] and Brazil [31], which emphasized the importance of supervising children during brushing. Concerns about dental and skeletal fluorosis were also low, at 4.6% and 2.2%, respectively, compared to 27.3% in Malaysia [32]. Studies from Riyadh [17] and China [15] revealed limited knowledge about fluoride’s side effects, though some Brazilian dental students (43.4%) [26] and Kerala dentists (60.7%) [33] showed greater awareness. This underscores the need for improved education on safe toothpaste use, particularly for vulnerable groups like young children.

This study has several limitations resulting from its design. Due to its cross-sectional nature, it is not possible to determine cause–effect relationships. In addition, the sample size, sampling method, and design may not represent all medical, dental, or pharmaceutical professionals in Croatia. This study may be affected by selection bias, as some individuals might have opted out of participation, potentially impacting the generalizability of the results. Furthermore, an imbalance in the gender distribution among participants was noted, with more women participating than men. Despite these limitations, medical, dental, and pharmaceutical professionals showed better knowledge and awareness of fluoride compared to the general population. 

Future research should take these limitations into account by using larger, more diverse samples and conducting longitudinal studies to investigate causal relationships. Additionally, qualitative methods like interviews or focus groups could offer deeper insights into fluoride perceptions across various professional groups. Finally, interventions to improve fluoride education and awareness should be implemented and evaluated to enhance the knowledge of both professionals and the general population. This study on perceptions of fluoride toxicity and oral hygiene products provides important insights for public health and policy by highlighting misconceptions and concerns. By addressing these issues, targeted education can improve public understanding of fluoride safety. For policymakers, this study offers guidance for refining guidelines and regulations to ensure a balanced approach that maximizes benefits while addressing safety concerns. Prioritizing larger, more diverse samples and longitudinal studies in future research will help us to explore causal relationships and evaluate interventions. Ultimately, improving understanding and awareness of fluoride will support better-informed public health practices and evidence-based policy decisions, leading to improved oral health outcomes and prevention practices.

## 5. Conclusions

In summary, this study provides important insights into the knowledge of and attitudes towards fluoride among health professionals and the general public in Croatia. While dental professionals showed a strong understanding of fluoride, all other groups, including medical and pharmaceutical professionals and the general public, showed only moderate knowledge. Remarkably, there were no significant concerns about the use of fluoride in any group, and there was no correlation between the level of knowledge and the level of concern about the potential risks of fluoride. Despite the moderate overall knowledge and lack of concern, some misconceptions and uncertainties were identified in the study, particularly regarding the role of fluoride in preventing early dental caries and the potential negative effects of fluoride use. Based on these findings, it is recommended that targeted public health measures and educational campaigns be implemented to dispel these misconceptions and improve knowledge about fluoride. Professional development programs should be revised to ensure that dental, medical, and pharmaceutical professionals receive comprehensive training on fluoride. In addition, increased public awareness through targeted outreach can help to clarify the benefits and risks of fluoride and ensure its safe and effective use in oral hygiene products. 

## Figures and Tables

**Table 1:** Predictors of a higher fluoride knowledge score in dental practice: sociodemographic and professional characteristics.

Characteristic		Total N = 1129	Fluoride Knowledge Score	
			Median (95% CI)	*p*-Values
Sex	Man	261 (23.1)	5.00 (3.75–4.41)	0.002 *
	Woman	868 (76.9)	5.00 (4.50–4.85)	
Age group (years)	18–30	598 (53.0)	5.00 (4.38–4.81) ^a^	≤0.001 *
	31–45	316 (28.0)	5.00 (4.59–5.15) ^b^	
	46–60	147 (13.0)	5.00 (3.75–4.57)	
	≥61	68 (6.0)	3.50 (2.71–3.96) ^a,b^	
Education level	High school	413 (36.6)	4.00 (3.57–4.10) ^a,b^	≤0.001 *
	Bachelor’s degree	92 (8.1)	4.00 (3.21–4.29) ^c,d^	
	Master’s degree	569 (50.4)	6.00 (4.92–5.31) ^a,c^	
	MSc/PhD	55 (4.9)	6.00 (4.45–5.80) ^b,d^	
Employment	Unemployed	46 (4.1)	2.00 (2.07–3.88) ^a^	≤0.001 *
	Student	300 (26.6)	5.00 (4.34–4.96) ^b^	
	Employed	735 (65.1)	5.00 (4.55–4.91) ^a,c^	
	Retired	48 (4.3)	2.00 (1.67–3.12) ^b,c^	
Field of employment/education	Dentistry	309 (27.4)	7.00 (6.26–6.65) ^a,b,c^	≤0.001 *
	Medicine	375 (33.2)	5.00 (4.01–4.47) ^a,d,e^	
	Pharmacy	176 (15.6)	5.00 (4.68–5.36) ^b,d,e,f^	
	General population	269 (23.8)	2.00 (2.13–2.73) ^c,e,f^	
Socioeconomic status	Above average	254 (22.5)	6.00 (5.07–5.63) ^a,b^	≤0.001 *
	Average	806 (71.4)	5.00 (4.24–4.61) ^b,c^	
	Below average	69 (6.1)	3.00 (2.17–3.54) ^a,c^	

Data are presented as the frequency (percentages) and median (95% CI). Statistical significance was tested using the Mann–Whitney or the Kruskal–Wallis one-way ANOVA test. The same lowercase superscript letter indicates a statistical difference between groups obtained by the Kruskal-Wallis test with post-hoc Dunn’s test * *p* ≤ 0.05.

**Table 2:** The attitudes and practices regarding fluoride usage and its application among participants.

Characteristic		Total (N = 1129)	Dentistry (N = 309)	Medicine (N = 375)	Pharmacy (N = 176)	General Population (N = 269)
Awareness of the effect of fluoride in reducing the frequency of caries	Yes	887 (78.6)	303 (98.1)	311 (82.9)	157 (89.2)	116 (43.1)
	No	242 (21.4)	6 (1.9)	64 (17.1)	19 (10.8)	153 (56.9)
Usage of fluoride toothpaste in daily tooth brushing routine	Yes	770 (68.2)	264 (85.4)	268 (71.5)	132 (75.0)	115 (42.8)
	No	139 (12.3)	32 (10.4)	40 (10.7)	19 (10.8)	48 (17.8)
	Not sure	220 (19.5)	13 (4.2)	67 (17.9)	25 (14.2)	106 (39.4)
Most commonly used chemical compound of fluoride	Aminofluoride (C₂₇H₆₀F₂N₂O₃)	60 (5.3)	39 (12.6)	12 (3.2)	6 (3.4)	3 (1.1)
	Sodium fluoride (NaF)	276 (24.4)	146 (47.2)	73 (19.5)	43 (24.4)	14 (5.2)
	Sodium monofluorophosphate (Na₂PFO₃)	28 (2.5)	15 (4.9)	4 (1.1)	7 (4.0)	2 (0.7)
	Tin fluoride (SnF₂)	15 (1.3)	7 (2.3)	3 (0.8)	3 (1.7)	2 (0.7)
	Calcium fluoride (CaF₂)	21 (1.9)	4 (1.3)	7 (1.9)	5 (2.8)	5 (1.9)
	Not using fluoride	55 (4.9)	15 (4.9)	16 (4.3)	10 (5.7)	14 (5.2)
	Do not know	674 (59.7)	83 (26.9)	260 (69.3)	102 (58.0)	229 (85.1)
Amount of fluoride in parts per million (ppm) in the toothpaste	High (above 1500 ppm)	24 (2.1)	15 (4.8)	6 (1.6)	2 (1.1)	1 (0.4)
	Standard (1000–1500 ppm)	292 (25.9)	183 (59.2)	52 (13.9)	38 (21.6)	19 (7.0)
	Low (500 ppm)	38 (3.3)	13 (4.2)	12 (3.2)	10 (5.7)	3 (1.1)
	Not using fluoride toothpaste	53 (4.7)	15 (4.9)	18 (4.8)	10 (5.7)	10 (3.7)
	Do not know	722 (64.0)	83 (26.9)	287 (76.5)	116 (65.9)	236 (87.7)
Usage of fluoride mouthwash	Daily	75 (6.6)	19 (6.1)	22 (5.9)	8 (4.5)	26 (9.7)
	Once a week	82 (7.3)	37 (12.0)	23 (6.1)	4 (2.3)	18 (6.7)
	Uncertain about fluoride content	100 (8.9)	5 (1.6)	42 (11.2)	17 (9.7)	36 (13.4)
	Uncertain about fluoride concentration	96 (8.5)	27 (8.7)	33 (8.8)	10 (5.7)	26 (9.7)
	Not using fluoride mouthwash	776 (68.7)	221 (71.5)	255 (68.0)	137 (77.8)	163 (60.6)
Usage of other sources of fluoride	Not using other sources	677 (60.0)	182 (58.9)	217 (57.9)	117 (66.5)	161 (59.9)
	Varnish	7 (0.6)	3 (1.0)	1 (0.3)	0 (0)	3 (1.1)
	Gel, cream or foam	21 (1.9)	5 (1.6)	5 (1.3)	2 (1.1)	9 (3.3)
	Milk	149 (13.2)	40 (12.9)	48 (12.8)	19 (10.8)	42 (15.6)
	Water	73 (6.5)	19 (6.1)	25 (6.7)	11 (6.3)	18 (6.7)
	Salt	114 (10.1)	28 (9.1)	39 (10.4)	20 (11.4)	27 (10.0)
	Supplements	14 (1.2)	5 (1.6)	5 (1.3)	1 (0.6)	3 (1.1)
	Restorative materials	25 (2.2)	8 (2.6)	9 (2.4)	2 (1.1)	6 (2.2)
	Other	4 (0.4)	2 (0.6)	0 (0)	1 (0.6)	1 (0.4)

Data are presented as the frequency (percentages).

**Table 3:** The distribution of responses to questions assessing participants’ knowledge of fluorides and their application in dentistry.

Question (Answer “Yes”)	Total (N = 1129)	Dentistry (N = 309)	Medicine (N = 375)	Pharmacy (N = 176)	General Population (N = 269)
Fluorides are common ingredients in pharmaceutical products for oral hygiene due to their recognized effect in the prevention of tooth decay.	728 (64.5)	255 (82.5)	236 (62.9)	131 (74.4)	106 (39.4)
Brushing twice a day with fluoride toothpaste lowers the risk of dental caries.	826 (73.2)	276 (89.3)	279 (74.4)	137 (77.8)	134 (49.8)
Fluoride can greatly help dental health by strengthening the tooth enamel, making it more resistant to tooth decay.	708 (62.7)	278 (90.0)	215 (57.3)	117 (66.5)	98 (36.4)
Fluoride prevents early dental caries by reducing bacterial metabolism, thus reducing acid production and hence demineralization.	493 (43.7)	195 (63.1)	155 (41.3)	83 (47.2)	60 (22.3)
Fluorides may be added to toothpastes in several different forms, such as sodium fluoride, sodium monofluorophosphate, tin fluoride, or in the form of different amines.	591 (52.3)	255 (82.5)	176 (46.9)	112 (63.6)	48 (17.8)
Depending on various factors, for example, concentration, frequency of use, and age of the user, fluoride can be detrimental or beneficial.	671 (59.4)	270 (87.4)	206 (54.9)	122 (69.3)	73 (27.1)
Fluoride toothpaste should be introduced with the eruption of the first tooth. Children under 3 years old are advised to use a small smear of toothpaste, while those aged 3 and older who can spit reliably should use a pea-sized amount.	594 (52.6)	244 (79.0)	177 (47.2)	105 (59.7)	68 (25.3)
Spitting out toothpaste without rinsing allows fluoride to remain in the mouth, continuing its effectiveness.	512 (45.3)	221 (71.5)	147 (39.2)	77 (43.8)	67 (24.9)

Data are presented as frequency (percentages).

**Table 4:** Evaluation of ingredient toxicity in oral hygiene products by participants.

Ingredient	Assessed Toxicity among Respondents				
	Total (N = 1129)	Dentistry (N = 309)	Medicine (N = 375)	Pharmacy (N = 176)	General Population (N = 269)
Fluoride	349 (30.9)	133 (43.0)	114 (30.4)	69 (39.2)	33 (12.3)
Alcohol	794 (70.3)	245 (79.3)	285 (76.0)	126 (71.6)	138 (51.3)
Triclosan	286 (25.3)	110 (35.6)	88 (23.5)	57 (32.4)	31 (11.5)
Parabens	566 (50.1)	181 (58.6)	221 (58.9)	110 (62.5)	54 (20.1)
Sodium lauryl sulfate	353 (31.3)	127 (41.1)	102 (27.2)	72 (40.9)	52 (19.3)
Chlorhexidine	269 (23.8)	83 (26.9)	108 (28.8)	53 (30.1)	25 (9.3)
Zinc salts	170 (15.1)	55 (17.8)	68 (18.1)	32 (18.2)	15 (5.6)
Propylene glycol	229 (20.3)	67 (21.7)	97 (25.9)	47 (26.7)	18 (6.7)
Artificial dyes	600 (53.1)	170 (55.0)	242 (64.5)	96 (54.5)	92 (34.2)
Sodium benzoate	277 (24.5)	89 (28.8)	108 (28.8)	50 (28.4)	30 (11.2)
Artificial sweeteners	569 (50.4)	154 (49.8)	224 (59.7)	90 (51.1)	101 (37.5)
Cocamidopropyl betaine	192 (17.0)	59 (19.1)	85 (22.7)	31 (17.6)	17 (6.3)
Hydrogen peroxide	438 (38.8)	144 (46.6)	170 (45.3)	85 (48.3)	39 (14.5)
Diethanolamine (DEA)	257 (22.8)	78 (25.2)	102 (27.2)	52 (29.5)	25 (9.3)
Cetylpyridinium chloride (CPC)	246 (21.8)	73 (23.6)	101 (26.9)	47 (26.7)	25 (9.3)

Data are presented as frequency (percentages).

## Data Availability

The data presented in this study are available on request from the corresponding author due to privacy and ethical reasons.
